# The pattern and characteristics of sexual assault perpetrators and survivors managed at a sexual assault referral centre in Lagos

**DOI:** 10.4102/phcfm.v10i1.1727

**Published:** 2018-11-15

**Authors:** Oluwajimi O. Sodipo, Ayoade Adedokun, Adedeji O. Adejumo, Olushola Oibamoyo

**Affiliations:** 1 Department of Family Medicine, Lagos State University Teaching Hospital, Nigeria; 2 Department of Community Health and Primary Health care, Lagos State University Teaching Hospital, Nigeria; 3 Department of Psychiatry, Lagos State University Teaching Hospital, Nigeria

## Abstract

**Background:**

There has presumably been an increase in cases of sexual assault in Nigeria; however, accurate data on the characteristics of the survivors and perpetrators are not readily available in most cases.

**Aim:**

To report the pattern and characteristics of sexual assault perpetrators and survivors managed at the Mirabel Centre, Lagos State University Teaching Hospital (LASUTH), Ikeja – a three-year review.

**Methods:**

A retrospective audit carried out in the sexual assault referral centre (SARC) – Mirabel Centre, LASUTH, Lagos State, Nigeria. A total of 2160 case files from July 2013 to January 2017 were assessed for age group of survivors and gender, among others.

**Results:**

A total of 2160 cases were analysed. The mean age of survivors was 13.9 (± 4.4) years with the 11–20 years age group having the highest number of survivors (48.4%) and the 0–10 years age group having 35.9% of survivors. Majority of the survivors (97.7%) were female. The majority of the perpetrators were known to the survivors with 10.3% being family members. Defilement (71.6%) was the most reported type of assault at the centre with rape (20.3%) being the second most common. The majority of the referrals to the centre were from the police (76.7%), while self-referrals made up 8% of referrals.

**Conclusion:**

Minors and adolescents formed the majority of the survivors. Defilement was the most reported form of sexual violence. There needs to be special intervention for better monitoring and protection of minors and youths against sexual assault. The establishment of more sexual assault referral centres in Nigeria will increase reportage and treatment of survivors.

## Introduction

Sexual assault has been defined as any sexual act, attempt to obtain a sexual act, unwanted sexual comments or advances, or acts to traffic, or otherwise directed, against a person’s sexuality using coercion, by any person regardless of their relationship to the survivor, in any setting, including but not limited to home and work.^1^ Sexual violence can include other forms of assault involving a sexual organ or object, including coerced contact between the mouth and penis, vulva or anus.^1^

It can also be defined as a sexual act committed against someone without that person’s consent.^2^

The incidence of sexual assault is on the increase worldwide.^1^ Available data show that nearly one in four women may experience sexual violence by an intimate partner^3^ and up to one-third of adolescent girls report their first sexual experience as being forced in many countries.^1^

Recent data from various countries such as Chile, Malaysia, Mexico, Papua New Guinea and the United States indicate that between one-third and two-thirds of all victims of sexual assault are aged 15 years or less.^4^

A study by the Centre for Disease Control (CDC) reported that 42.2% of female rape victims were first raped before age 18, and about 30% of male rape victims were first raped when they were age 10 or younger.^5^

In the Democratic Republic of Congo, 16% of women reported having sex against their will.^6^ Another study among female university students in Ethiopia reported 14.3% of them having experienced rape since being admitted into the university.^7^

In Nigeria, the prevalence of rape from facility-based studies varies from 2.1% – 5.6%.^8,9,10^ A study in Ibadan showed that 15% of young females reported forced penetrative sexual experience,^11^ while 13.8% of female Maiduguri students reported the same.^12^

In Nigeria, a poll by the National Opinion Institute in 2014 revealed that 31% of respondents knew a victim of child rape, while close family members, friends and neighbours were identified by over a third of respondents as perpetrators.^4^

Some of the risk factors for sexual assault include being young, consuming alcohol or drugs, previous history of rape or sexual abuse, students, having multiple sexual partners and poverty, among others.^13^

Sexual violence could involve single or multiple perpetrators, with a study carried out in Johannesburg, South Africa, reporting that one-third of the cases presenting to a women’s clinic had been gang rapes and the survivors knew the perpetrators.^14^

The health consequences of sexual assault include physical injuries, unwanted pregnancies, unsafe abortions and sexually transmitted diseases, including human immunodeficiency virus (HIV).^15^ There could also be short-term and long-term psychological complications like depression and suicide.

The Mirabel Centre is the first sexual assault referral centre (SARC) in Nigeria established in 2013 and is situated in the Lagos State Teaching Hospital, Lagos State.

The aim of this retrospective audit was to ascertain the pattern and characteristics of sexual assault perpetrators and survivors managed at the Mirabel Centre, Lagos State University Teaching Hospital (LASUTH), Ikeja, over a 3-year period with a view to improving the management of survivors of sexual assault.

## Methodology

### Study design

A retrospective review of clients’ records seen from 01 July 2013 to January 2017 was conducted to obtain the age of clients presenting to the centre, their gender, source of referral to the centre, multiplicity of perpetrators, clinical services rendered and relationship between the perpetrator and the survivor.

### Setting

The Mirabel Centre is the first SARC in Nigeria. It is a collaborative effort between a non-governmental organisation (partnership for justice) and the Department of Family Medicine, LASUTH, Ikeja, Lagos State, Nigeria. It was opened on 01 July 2013 and provides comprehensive care at no cost to all survivors of sexual assault. The staff population is a mixture of consultant family physicians, resident doctors, nurses and other support staff, all of whom have undergone training on forensic medical examination.

### Operative definitions^16^

- Survivor: any person who was sexually assaulted, defiled, raped or attempted to be raped and was not killed in the process.
- Perpetrator: any person who commits sexual assault, rape or defilement.
- Rape: unlawful sexual intercourse or penetration of a male or female (≥ 18 years old) without consent.
- Defilement: any person who has sexual intercourse or penetrates a child (< 18 years of age).
- Sexual assault: defined as touching, sending sexually explicit images or making sexually explicit comments towards another person without his or her consent.
- Attempt to commit rape and sexual assault by penetration: Attempts to commit the offence of rape or sexual assaults by person with a part of the body or anything else, without the consent of the person.

### Data collection

The Mirabel Centre requires that clients compulsorily sign consent forms allowing the anonymous use of their information and treatment, while minors and children have the consent forms signed on their behalf by parents or guardians. All case files of clients of the Mirabel Centre who had signed consent forms allowing the anonymous use of their data were analysed. Case files with incomplete information (such as age of client and source of referral and details of the perpetrator) were excluded.

For the period outlined, 2213 clients had presented to the centre, 32 (1.45%) of these did not have signed consent forms on data use and 21 (0.95%) did not have complete information. A total of 2160 (97.6%) case files met the criteria for analysis. The age of clients presenting to the centre, their gender, source of referral to the centre, multiplicity of perpetrators, clinical services rendered and relationship between the perpetrator and the survivor were analysed.

### Analysis

Results were analysed using the SPSS 19 (Statistical Package for the Social Sciences [SPSS] Inc., Rochester, MN, US). Statistics were presented in tabular and descriptive forms.

## Ethical considerations

Ethical clearance was obtained from the ethical committee of LASUTH, Ikeja (Registration number NHREC04/04/2008, Reference number LREC.06/10/793). Confidentiality was maintained as data were anonymised; therefore, the names of sexual assault cases could not be linked to the data.

## Results

The mean age of the survivors was 13.9 (±4. 4) years. The 11–20 years age group had the highest number of survivors with 1045 (48.4%), while the above 30 years age group had the lowest number with 65 cases (3%). Majority of the survivors (2110, 97.7%) were females, with males making up only 2.3% as shown in Table 1.

**TABLE 1:** Age distribution and gender of the survivors.

Variable	Frequency (*N* = 2160)	%
**Age group (years)**		
0–10	775	35.9
11–20	1045	48.4
21–30	275	12.7
> 30	65	3.0
**Gender**		
Female	2110	97.7
Male	50	2.3

Note: Mean age: 13.9 years (±4.4)

*Source*: Data obtained from Mirabel Centre, Lagos State University Teaching Hospital, LASUTH, Ikeja

About three-quarters (1588, 73.5%) of the perpetrators were known to the survivors with a tenth (222, 10.3%) being family members. In the majority of assaults reported at the centre (1877, 86.9%), the perpetrators were alone, while 237 (11%) cases reported involved multiple perpetrators as shown in Table 2. Defilement was the most reported type of assault at the centre (1547, 71.6%), with rape (438, 20.3%) being the second most common. Others included sexual assault and attempt to commit rape as shown in Table 2.

**TABLE 2:** Showing the characteristics of perpetrators and clinical services provided.

Characteristics of perpetrator and clinical services	Frequency	
	*N*	%
**Relationship of perpetrator**		
Unknown	350	16.2
Non-family	1588	73.5
Family	222	10.3
**Number of perpetrators**		
Single	1877	86.9
Multiple	237	11.0
Unknown	46	2.1
**Type of incident**		
Rape	438	20.3
Attempt to commit rape or penetration	41	1.9
Sexual assault	125	5.8
Defilement	1547	71.6
Others (physical assault)	9	0.4
**Source of referral**		
Police	1657	76.6
LASUTH	129	6.0
NGO	53	2.5
Other government agencies	148	6.9
Self-referral	173	8.0
**Clinical services provided**		
Counselling	2045	94.7
Medical services	2111	97.7
Forensic report to the police	1657	76.7

*Source*: Data obtain from Mirabel Centre, LASUTH, Lagos state University Teaching Hospital

NGO, non-governmental organisation.

The majority of the referrals to the centre were from the police (1657, 76.6%). Self-referral by survivors (173, 8%) and referrals from other departments in the hospital (129, 6%) were other sources of referral to the centre as shown in Table 2. Counselling (2045, 94.7%) and medical treatment (2111, 97.7%) were instituted in a majority of the survivors, while about three-quarters of the survivors (1657, 76.7%) had forensic medical reports prepared for use by the police as shown in Table 2.

## Discussion

This study showed that the average age of survivors was 13.9 years, with children and adolescents (0–20 years) accounting for a majority of survivors. A similar local study by Akinlusi et al. in Lagos also reported that the adolescent age group (10–19 years) accounted for the majority (44.6%) of the cases, followed by children less than 10 years (39.0%). The finding is also in keeping with reported observations from other parts of the world that children and adolescents account for majority of sexual assault cases.^4,15,17,18,19,20^

A possible reason for this observation is that children are less likely to offer resistance to their assailants and may be too young to understand what constitutes sexual assault.^21,22^ It has also been observed that young survivors delayed presentation because they were scared of what relatives would say about alleged sexual assault.^23^

Our finding that majority of the survivors were female is in keeping with previous studies which have reported that majority of sexual assault survivors are female.^15,24,25^ Studies carried out in India,^18^ Bangladesh^26^ and Uganda^27^ have also reported low rates of sexual assault in male gender as shown in this study. It is, however, important that as preventive measures are directed towards females, male survivors and males at risk are not neglected.

The majority of survivors (83.5%) were reported to know their perpetrators. Vetten et al. also reported that in 84% of rapes, perpetrators known to survivors carry out the act, especially if involving young survivors.^28^ Another study in South Africa reported that 67.6% of the survivors knew the perpetrators, highlighting the fact that sexual assault is usually committed by people known to the survivors.^20^ This finding is also documented in studies elsewhere in Nigeria where the perpetrators of the sexual assaults were known to the survivors either as acquaintances or as authority figures.^8,9,15,27,28^ The psychological complications of sexual assault carried out by perpetrators known to survivors could result in more psychologically damaging outcomes than being assaulted by a stranger.^20^ Such incidents could also destroy the ability of survivors to trust people.^21^

In the majority of assaults reported at the centre, the perpetrators were single. This finding is similar to that reported in Sokoto State, Nigeria, that 91.2% of survivors were assaulted by only one perpetrator.^24^ Similarly, reports from medico legal clinics in Johannesburg, South Africa, following rape found that majority involved one perpetrator.^14^

Defilement was the most common form of sexual assault reported in this study. This is in keeping with the observation that the majority of the survivors were children and adolescents. The finding is in keeping with other studies worldwide and Nigeria that have reported minors form a majority of sexual assault survivors.^15,24^

About three-quarters of the referrals to the centre were from the police, while self-referrals and referrals from other government agencies were additional sources of referral. A study in Sokoto State, Nigeria, reported that the majority of cases (58.8%) were reported to law enforcement.^24^ This may not be unconnected with the fact that most sexual assault survivors are children and adolescents, and hence, their parents would take the decision to report the incident to the police.^30^ Studies have also reported higher likelihood of arrests by law enforcement agents in juvenile assaults.^10,29,30,31^

The finding that that majority of the clients had forensic reports prepared is because of the fact that clinical staff of the Mirabel Centre have been trained on forensic medical examination which would help in criminal prosecution of the perpetrators. The Mirabel Centre being the first SARC in Nigeria and Lagos would also account for the multiplicity of agencies who referred survivors for examination and treatment.

## Limitations

This study involved only one SARC; hence, its findings may not be applicable to the general population. There could also be recall bias from survivors who were children.

## Conclusion

Minors and adolescents formed a majority of the survivors, and defilement was the most reported form of sexual assault. There needs to be special intervention for better monitoring and protection of minors and youths against sexual assault. Increased public awareness and establishment of more sexual assault referral centres in Nigeria will go a long way in increasing reportage and providing treatment for survivors.
